# Aseptic nonunion of the tibia treated by plating and bone grafting: retrospective study about 40 cases

**DOI:** 10.1186/s13018-022-03216-z

**Published:** 2022-06-21

**Authors:** Khalid Chmali, Mohammed ElIdrissi, Hatim Abid, Abdelhalim ElIbrahimi, Mohamed Berraho, Abdelmajid ELMrini

**Affiliations:** 1 Surgery Department, CHP La Marche Verte, Boulemane, Morocco; 2 Trauma-Orthopedic Department B4, CHU Hassan II, Fez, Morocco; 3 Laboratory of Epidemiology, Clinical Research and Community Health, USMBA–FMPDF, Fez, Morocco

**Keywords:** Aseptic nonunion, Tibia, Bone graft, Bone plate, Retrospective study

## Abstract

**Objective:**

The objective of this study was to evaluate the clinical and radiological results of the treatment of aseptic nonunion of the tibia by plating and bone grafting.

**Material and Methods:**

This retrospective study included 40 patients with aseptic nonunion of the tibia, treated in the Trauma-Orthopedic department B4 of CHU Hassan II in Fez-Morocco. The average age was 41 years (range 25–60 years). The initial fractures were in the middle third of the tibia for the majority of our patients. We used the ASAMI criteria to assess the results.

**Results:**

We found 45 patients with aseptic nonunion of the tibia who were treated by the same surgical team and followed in postoperative consultation for a fixed period of 10 months. Three patients lost to follow-up and two patients refused the treatment. In 37 patients (92.5%), union was obtained after a mean delay of 4.3 months (range 3–7 months). The average time from initial treatment to treatment for nonunion was eight months (range 6–10 months). According to the ASAMI classification, bone results were excellent in 26, good in 8, fair in 3 and poor in 3; functional results were excellent in 10, good in 16, fair in 11 and poor in 3.

**Conclusions:**

Our study suggests that the combination of screwed plate and autograft in the treatment of aseptic nonunion of the tibia has provided satisfactory results. A well-codified management of the initial fracture remains the gold key to prevent the occurrence of pseudarthrosis.

## Introduction

A uniform definition of pseudarthrosis does not exist. However, in the literature, authors agree on the notion of the duration of consolidation [1]. Some authors define pseudarthrosis as a fracture that has not healed after six months of treatment or repeated surgical interventions [1]. Others define pseudarthrosis as a cessation of the healing process, usually six to nine months after the trauma, in the absence of consolidation [1]. Some authors use Antonova's definition of pseudarthrosis as a fracture that cannot heal without additional interventions, surgical, or otherwise, between 6 and 9 months [1]. It occurs in 5–10% of cases [2] and can reach 30% [3–6]. It is clinically estimated by the persistence of pain and residual mobility in the fracture site**.** In the leg, aseptic nonunion is a topical problem and one of the most feared complications due to its serious consequences. It can be secondary to orthopedic or surgical treatment, and several risk factors are involved in its evolution [7–10]. Many treatments are described in the literature, the choice of which depends on the character of the nonunion as well as the quality of alignment of the fragments, and the chances of a satisfactory end result. The objective of this work is to evaluate the clinical and radiological results of the treatment of aseptic nonunion of the tibia by screw plate associated with a cortico-cancellous bone autograft.

## Materials and methods

This is a retrospective study involving 40 patients, treated for aseptic nonunion of the tibia between January 2015 and January 2020, at the Trauma-Orthopedic department B4 of CHU Hassan II in Fez. The inclusion criteria referred to the existence of an aseptic nonunion of the tibia treated by screw plate associated with an autologous bone graft. All our patients were treated by the same surgical team and followed in consultation for a period of 10 months. We excluded from our study: septic nonunion, metaphyseal–epiphyseal nonunion, nonunion on pathological fracture and nonunion treated by other surgical techniques, as well as patients with an incomplete medical file, or having refused treatment. The data were collected by doctors from the Trauma-Orthopedic department B4, using an operating sheet filled in from the medical records of the patients included in the study. We used the AO classification on the initial x-rays to classify tibial fractures. Radiographs of delayed union showed no bone union between three and six months after initial treatment of the tibia fracture, while pseudarthrosis (PSA) tibial radiographs showed nonunion after six months. On these X-rays, we analyzed the initial treatment of the tibial fracture and the type of PSA. The indication for our surgical treatment was based on the presence of clinical signs of PSA (pain with or without mobility of the fracture site) and radiological (absence of bone union) after six months of the initial treatment. The surgical treatment comprises an osteosynthesis by a traditional screwed plate associated with a cortico-cancellous autograft taken from the anterior ipsilateral iliac crest. The approach used was the anterolateral leg approach. Monopodal support was prohibited for 2 months and passive functional rehabilitation was started early with isometric contractions. All the patients were reviewed in consultation with an evaluation of the mobility of the knee and ankle, an assessment of the pain on a visual analogue scale, with a study of the morbidity of the engraftment site. The follow-up radiological assessment included a frontal and lateral view of the tibia. Consolidation was taken for obtained given the absence of graft necrosis and the existence of bone bridges between the two ends of the PSA site. To analyze the evolution of the aseptic nonunion of the leg after treatment, we used the ASAMI classification (Table 1).

**Table 1:** Classification of the results according to the modified ASAMI protocol [11]

	Bone result	Functional result
Excellent	Bone union, no infection, deformity < 7°, LLD (lower limb discrepancy) < 2.5 cm	Ability to perform previous activities of daily living (ADL), No pain or mild pain, No limp, No soft tissue sympathetic, dystrophy knee or ankle joint contracture < 5°, Loss of ankle or knee motion < 15°
Good	Bone union, failure to meet one of the other criteria	Almost all ADL with minimal difficulty, No pain or mild pain, failure to meet one of the other criteria
Fair	Bone union, failure to meet two of the other criteria	Most ADL with minimal difficulty No pain or mild pain, failure to meet two of the other criteria
Poor	Nonunion or refracture, failure to meet three of the other criteria	Significantly limited ADL, Significantly pain requiring narcotics, failure to meet three of the other criteria

## Results

**Fig. 1:**
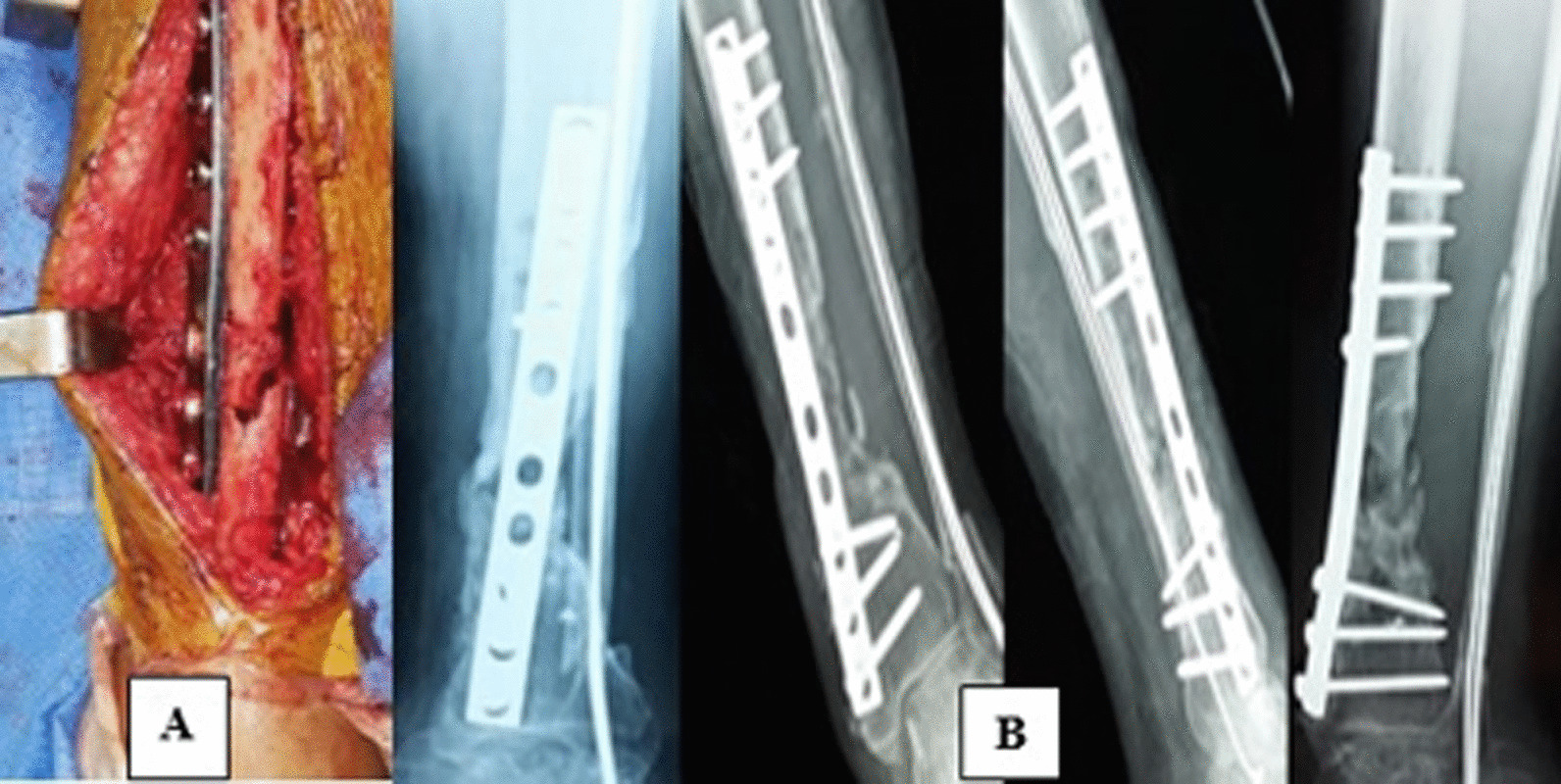
**A** Placement of the cortico-cancellous graft. **B** Radiological result at 4 months

**Fig. 2:**
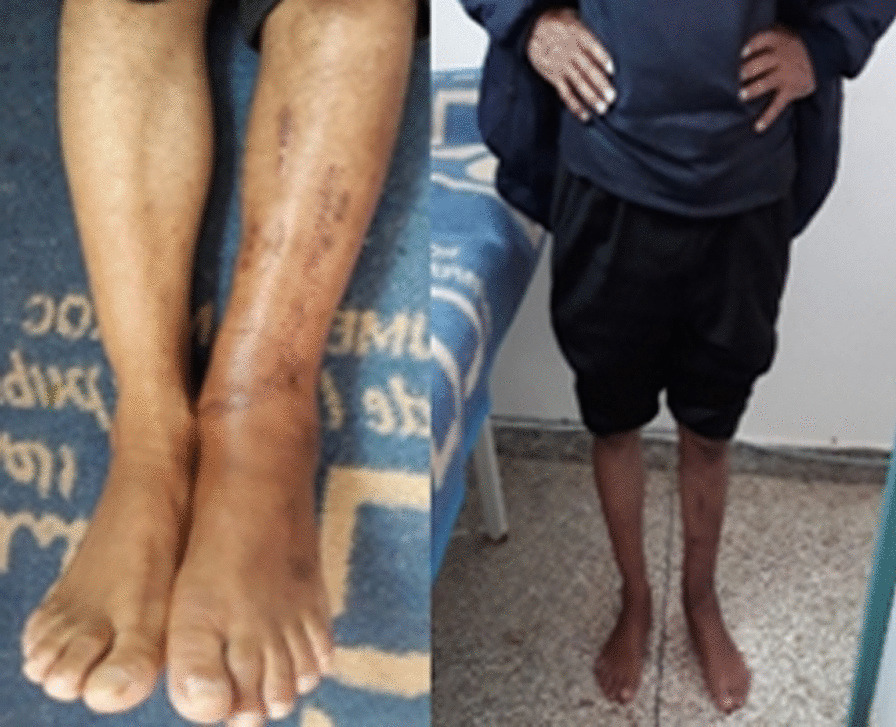
Clinical result at 4 months

Out of 546 leg fractures collected during the study period from January 2015 to January 2020, 45 cases (8.2%) were complicated by aseptic nonunion of the tibia. Of these 45 cases, 5 patients were excluded (3 lost to follow-up and 2 refused treatment). There remained 34 men (85%) and 6 women (15%) (Table 2). The average age of our patients was 41 years (range 28 to 59 years). Public road accidents were the main etiology found in 34 cases (85%), and there were also 3 cases (7.5%) of sports accidents (football): 2 cases (5%) of a fall from a high place and one case (2.5%) of aggression (Table 2). The initial open fracture was found in 35 cases (87.5%), with a predominance of type III according to Gustilo's classification. No initial vasculo-nerve damage was present in the patients studied. The study of the line of the initial fracture according to the AO classification of diaphyseal fractures of the tibia showed: eight fractures A1 (spiroid), eleven A2 (oblique), five A3 (transverse), seven B1 (torsion wedge) and nine C3 (comminuted) (Table 2). Initial bone loss was observed in 23 cases (57.5%). At diagnosis, all patients were in pain with an average visual analogue scale of 8/10 (range 5 to 10). Mobility of the nonunion site was observed in 10 patients (25%). The initial treatment consisted of 21 external fixation (52.5%), 3 screw plates (7.5%), 16 intramedullary nailing (40%) (Table 2). We found nine cases (22.5%) of technical errors in the initial treatment in our series. The most common site of aseptic nonunion of the tibia was the middle 1/3 of the tibia in 22 patients (55%). The nonunion-type study showed 4 eutrophic PSA (10%), 24 atrophic PSA (60%) and 12 hypertrophic PSA (30%) (Table 2). The average time from initial treatment to treatment for nonunion was eight months (range 6–10 months). The immediate postoperative consequences were simple in 36 patients (90%), with 4 cases (10%) of superficial infection in patients initially treated with an external fixator, controlled by local care and appropriate antibiotic therapy. We noted no case of pressure ulcers or phlebitis, and all the intraoperative bacteriological samples came back negative. Radiological analysis revealed two vicious calluses of the tibia (1 callus in varus of 5° and 1 callus in recurvatum of 10°) which were functionally tolerable. In 37 patients (92.5%), union was obtained after a mean delay of 4.3 months (range 3–7 months), while 3 patients (7.5%) had a persistence of nonunion which resulted in need for multiple interventions. The criteria for recovery were absence of pain and radiological consolidation of the nonunion site. At the last follow-up, the mean mobility of the knee and ankle improved (Table 3). The results of our patients (Table 4) were analyzed according to the ASAMI score (Figs. 1 and 2).

**Table 2:** Radio-clinical data of 40 patients

Data	Number of patients
*Etiologies*	
Road accidents	34
Sports accident (football)	3
Fall from a high place	2
Aggression	1
*Sex*	
Male	34
Female	6
*Type of fracture (AO classification)*	
Type A1	8
Type A2	11
Type A3	5
Type B1	7
Type C3	9
*Initial treatment*	
External fixator	21
Screw plate	3
Intramedullary nailing	16
*Type of pseudarthrosis*	
Eutrophic	4
Atrophic	24
Hypertrophic	12

**Table 3:** Pre- and postoperative mobility

Average amplitudes	Knee		Ankle	
	Flexion	Extension	Dorsiflexion	Plantar flexion
Preoperative	100° (90–110°)	Average deficit of 10°	10° (0–20°)	20° (10–40°)
Postoperative	110° (90–120°)	Average deficit of 5°	20° (10–30°)	30° (20–50°)

**Table 4:** Bone and functional results of the 40 patients

Results	Bone		Functional	
	Number of patients	(%)	Number of patients	(%)
Excellent	26	65	10	25
Good	8	20	16	40
Fair	3	7.5	11	27.5
Poor	3	7.5	3	7.5
Total	40	100	40	100

## Discussion

This retrospective study has enabled us to show that the treatment of aseptic nonunion of the tibia by screw plate associated with an autograft bone constitutes an effective therapeutic alternative with very encouraging functional and radiological results. The high incidence of aseptic nonunion on the tibia can be explained by poor tissue coverage and therefore poor blood circulation [6]. Several factors may be involved in the occurrence of aseptic tibial nonunion [7–10]. One of the main factors favoring aseptic nonunion of the tibia remains the technical errors during the initial treatment: persistent interfragmentary gap, insufficient volume of screw plate or insufficient number of screws, insufficient of size or diameter of intramedullary nail and failure of the fixation. We found nine cases (22.5%) of these technical faults in our series. We also noted a clear male predominance (85%), like several series in the literature [12–16], and this peculiarity is thought to be due to male exposure to violent trauma. The average age of our patients was 41 years (range 28–59 years) which matches the data of the series [12–16], and this age group corresponds to a period when there is a lot travel and professional activity. Road accidents are the main cause (88.4%) of initial fractures [17]; in our patients, this causal agent represents 85% of cases. In our series, the initial open fracture was found in 35 patients (87.5%) with a predominance of type III according to the Gustilo classification; we observed: 10 patients of type I, 4 patients of type II, 18 patients of type IIIA and 3 patients of type IIIB, which agrees with the data in the literature [14–16], and skin opening can be correlated with the decrease in bone healing power as well as the initial bone loss which was observed in 57.5% of patients. On the clinical level, the main symptom is the pain associated or not with a functional impotence or an instability, and this pain is localized at the level of the fracture site, appearing with the mobility or the weight bearing [18]**.** All of our patients were painful at the PSA focus, which was mobile in 25% of patients. The standard face and profile X-ray remains a simple, noninvasive examination that makes it possible to make the radiological diagnosis of aseptic nonunion of the tibia and to specify its type and site. Seydou [12] and Bouzidi [19] reported that the initial fracture was located at the distal 1/3 of the leg and represented 36% of cases. In our series, the most common site of aseptic nonunion of the tibia was the middle 1/3 of the leg (55%). Complex fractures make bone healing more difficult, given the comminution that leads to devascularization of one or more fragments and makes mechanical stabilization difficult [20]. Like several series in the literature [21, 22], it was found in our series that atrophic aseptic nonunion is the type most frequently found with a rate of 60%. 52.5% of our patients were initially treated with external fixator (81.8% of patients had external fixator according to [14]), while 40% of our patients had intramedullary nailing (16% of patients had intramedullary nailing according to [23]). [24] presented 2 cases of infection with an external fixator with 1 case of skin necrosis, and we found 2 cases (5%) of superficial infection in patients initially treated with an external fixator, controlled by local care and appropriate antibiotic therapy. Several treatments are proposed for the management of aseptic nonunion of the leg, the goal of which is to obtain as soon as possible a consolidation of the site of PSA with correction of the axis and the length of the bones, as well as the conservation satisfactory function of the lower limb [18]. Internal fixation by intramedullary nailing is a so-called closed hearth technique [25], allowing the stabilization of the site without periosteal removal. Intramedullary nailing will find its place in hypertrophic PSA without significant alignment disturbance, and the supply of the reamer, equivalent to an in situ graft, promotes the formation of the periosteal callus [26]. The major risk of this treatment is contamination of the entire bone in cases of septic nonunion or when placing a nail after an external fixator [26, 27]. External fixation allows stable fixation, compression of the focus and correction of axis disorders [26]. Screwed plate osteosynthesis makes it possible to revive the bone ends, permeabilized the medullary canal and ensures stable osteosynthesis with correction of defective postures [28–30]. This is the only technique making it possible, during the same procedure, to ensure good stabilization of the PSA focus, while providing an osteogenic complement essential for consolidation and to take bacteriological samples from the PSA site. There are several means of stimulating osteogenesis, including cortico-cancellous autograft mainly taken from the iliac bone and which has a role in inducing the consolidation and filling of a bone defect [28], and it can be considered as the gold standard for the treatment of aseptic nonunion of the tibia, [31, 32], with a consolidation rate of 80–85% according to [33]; in our series it is 92.5%. We report potential morbidity at the donor site, quantified in the literature up to 30% [31] (hematomas, chronic pain, infection). Our results are therefore comparable with those of different authors, and we had a minimal septic risk and a satisfactory consolidation rate (92.5%), so this method (screwed plate and cortico-cancellous autograft) constitutes from our point of view an excellent alternative therapeutic for aseptic nonunion of the tibia.

## Limitations of the study

The main limitation of our work is its retrospective aspect, which may have been conducive to recall bias. The limited number of cases (40 patients) reflects the rarity of this pathology, but does not allow a multivariate analysis to be carried out and obliges us to remain descriptive. The subjective analysis of pain based on the visual analogue scale may cause confusion bias. Another possible bias relates to an erroneous evaluation of the results (patients lost to follow-up or who did not accept the treatment) which may underestimate the importance of the screw plate with the bone autograft in the treatment of aseptic nonunion of the tibia which, according to our results, was considerable.

## Conclusion

Aseptic nonunion of the tibia is a serious complication of leg fractures, and it remains very disabling with heavy socioeconomic and professional repercussions. Through this work, we have found that the combination between the screw plate and the cortico-cancellous autograft is the most appropriate, allowing a 92.5% consolidation rate with 65% good and excellent functional results. However, a well-codified initial management should allow better results to be obtained regardless of the treatment considered.

## Data Availability

Not applicable.
